# Detection of Postural Control in Young and Elderly Adults Using Deep and Machine Learning Methods with Joint–Node Plots

**DOI:** 10.3390/s21093212

**Published:** 2021-05-05

**Authors:** Posen Lee, Tai-Been Chen, Chi-Yuan Wang, Shih-Yen Hsu, Chin-Hsuan Liu

**Affiliations:** 1 Department of Occupation Therapy, I-Shou University, No. 8, Yida Rd., Jiaosu Village, Yanchao District, Kaohsiung 82445, Taiwan; 2 Department of Medical Imaging and Radiological Science, I-Shou University, No. 8, Yida Rd., Jiaosu Village, Yanchao District, Kaohsiung 82445, Taiwan; ctb@isu.edu.tw (T.-B.C.); wang1031@gmail.com (C.-Y.W.); e414888@gmail.com (S.-Y.H.); 3 Institute of Statistics, National Yang Ming Chiao Tung University, No. 1001, University Rd., Hsinchu 30010, Taiwan; 4 Department of Radiology, Zuoying Branch of Kaohsiung Armed Forces General Hospital, No. 553, Junxiao Rd., Zuoying District, Kaohsiung 81342, Taiwan; 5 Department of Information Engineering, I-Shou University, No. 8, Yida Rd., Jiaosu Village, Yanchao District, Kaohsiung 82445, Taiwan; 6 Department of Occupational Therapy, Kaohsiung Municipal Kai-Syuan Psychiatric Hospital, No. 130, Kaisyuan 2nd Rd., Lingya District, Kaohsiung 80276, Taiwan

**Keywords:** postural control, deep learning, machine learning, joint–node plot

## Abstract

Postural control decreases with aging. Thus, an efficient and accurate method of detecting postural control is needed. We enrolled 35 elderly adults (aged 82.06 ± 8.74 years) and 20 healthy young adults (aged 21.60 ± 0.60 years) who performed standing tasks for 40 s, performed six times. The coordinates of 15 joint nodes were captured using a Kinect device (30 Hz). We plotted joint positions into a single 2D figure (named a joint–node plot, JNP) once per second for up to 40 s. A total of 15 methods combining deep and machine learning for postural control classification were investigated. The accuracy, sensitivity, specificity, positive predicted value (PPV), negative predicted value (NPV), and kappa values of the selected methods were assessed. The highest PPV, NPV, accuracy, sensitivity, specificity, and kappa values were higher than 0.9 in validation testing. The presented method using JNPs demonstrated strong performance in detecting the postural control ability of young and elderly adults.

## 1. Introduction

Postural control is a complex motor function derived from several integrated neural components, including sensory and movement strategies, orientation in space, biomechanical constraints, and cognitive processing [1]. It is also the ability to build up posture against gravity and ensure that balance is maintained. Force plates are frequently used to measure balance [2,3]. Force plate equipment and motion analysis machines allow therapists to accurately describe the center of gravity (COG) location, center of body mass (COM) position, center of pressure (COP) displacement, and kinematics of movement strategies for balance. COG is the average location of the weight of an object. COM is the average position of all the parts of the body, weighted according to mass. However, the movements of body parts make assessing postural control by measuring average location, position, or displacement (COG, COM, COP) challenging [4]. Measuring postural control is difficult because postural changes may occur as a result of slight movements that are difficult to detect through simple observation by human eyes [1]. Observational balance measures such as the Berg Balance Scale are used to evaluate balance. However, they evaluate performance and not balance movement strategies. The assessment scales used by therapists tend to be subjective, and their reliability and sensitivity can be limited [5]. Measurements of postural control should identify how stably or quickly a subject performs or maintains an equilibrium position and the appropriateness and efficiency of movement strategies used to achieve or maintain the equilibrium position. Objective measures of postural control using computerized systems can allow more sensitive, specific, and responsive assessments in clinical practice.

Microsoft Kinect is a popular human motion capture tool. Kinect cameras are useful, as they provide joint center position data directly without additional processing of depth or image data [6]. Recent evidence suggests that Kinect may enable low-cost balance assessments and gait analyses [7,8,9,10,11,12,13,14,15,16]. The Kinect device has been reported to have validity for the evaluation of spatiotemporal gait parameters [17]. Kinect’s kinematic information is generally accurate enough for ergonomic assessments [18]. Postural control is the coordination of multiple joints to maintain postural stability, and the device can be used to collect large amounts of joints data to explore the coordinated relationships among the joints of the whole body during the maintenance of postural control [19]. Kinect’s kinematic parameters follow joint trajectories and, thus, can be used as a tool for measuring spatiotemporal aspects of postural control. Recent studies have demonstrated that 3D motion analysis of data from the Kinect motion capture system can be used in clinical assessments of coordination and balance and could potentially be used to monitor gross motor performance and assess motor function [20,21,22]. However, most studies have explored the displacement of COP, COM, or COG or the kinematics of body segments [7,11,13,23,24,25,26], whereas few have endeavored to classify the quality of postural control or measure slight differences in similar situations of postural control.

Neural networks have advanced at a remarkable rate, and they have practical applications in various industries, including the medical and health care industry [27,28,29,30]. Deep learning has major applications in medical diagnosis, classification, and prediction, including but not limited to health informatics [31] and biomedicine analysis [32]. Other uses of deep learning in the medical field are in medical image segmentation, registration, and detection of various anatomical regions of interest, such as in magnetic resonance imaging [33], ultrasound [34], and radiography [35]. The clinical use of images from digital cameras or depth sensors combined with deep and machine learning has promise for postural control assessment, body motion assessment, and fall detection. In one study, skeleton joints data from Kinect were used to determine human balance states, and a fall prediction algorithm based on recurrent neural networks and unbalanced posture features was proposed [36]. One fall detection method based on 3D skeleton data obtained from Kinect employed long short-term memory networks [37]. One study investigated the extent to which such deep learning–based systems provide satisfactory accuracy in exergame-relevant measures; a deep learning–based system was reported to perform as well as the gold standard system in the detection of temporal variations [38]. In one study, a long short-term memory recurrent neural network was used in a supervised machine learning architecture and a novel deep learning–refined kinematic model with good kinematic accuracy for upper limb functional assessment was developed [39]. Therefore, Kinect’s image information combined with machine and deep learning can be used to develop an effective limb functional assessment system for medical diagnosis or therapeutic evaluation.

Convolutional neural networks (CNNs) are the most widely represented class in deep learning and medical image analysis [27,28]. Deep learning methods are useful for extracting various image features, whereas machine learning approaches are efficient, rapid, and quantitative and can be used to build classification methods for numerous predictors. Hence, a combination of deep and machine learning methods was employed in this study. 

Objective measurements of postural control made with a computerized system using Kinect combined with machine and deep learning can enable sensitive postural control assessment in clinical practice. Such a system might effectively classify the quality of postural control or identify minute differences between cases of similar postural control. This study is the first to combine joint node motion information with machine learning to extract joint node trajectory features and to use deep learning to classify postural control stability according to joint node trajectory patterns. This work had a twofold aim: to extract joint node trajectory plot features in order to explore the relative motion and to classify the stability of postural control according to joint node trajectory patterns.

The remainder of the paper is organized as follows. The research methodology is described in Section 2. The experimental results are presented in Section 3. The proposed features for assessing postural control performance and the joint–node plot (JNP) are discussed in Section 4. Section 5 presents the conclusion and proposes future research directions.

## 2. Materials and Methods

### 2.1. Experimental Design in Young and Elderly Adults

**Figure 1:**
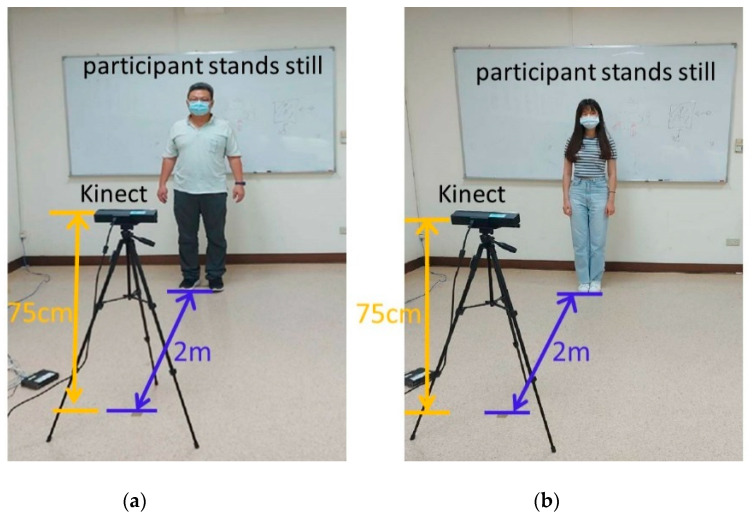
The Kinect device was placed 75 cm above the floor and 2 m in front of the participants. The participants stood still (with arms at their sides) in a comfortable stance for 40 s. The participants were defined as (**a**) elderly adults (experimental group) or (**b**) young adults (control group).

The experimental group was composed of elderly people who had a medical history and disabilities in daily life. They resided in a nursing home. In general, they might or could be regarded as a poor postural control group. In addition, the young adults had no medical history or any tremor problems. Therefore, the young group might or could be regarded as the control group. The study was conducted at a nursing home and on a college campus. Participants were recruited by a clinic nurse and study staff. To be included, participants had to meet the following criteria: be adults (>20 years old) to rule out developmental problems; have no restriction on physical activity; have no lower-limb discomfort and be able to maintain a double-leg stance with both eyes open for at least 40 s; and be willing to provide consent to participate in the study. The selected participants underwent the Mini-Mental State Examination (MMSE), Barthel Index (BI), and Berg Balance Scale (BBS) examinations in both young and elderly groups (Table 1). The young participants got full marks in the MMSE, BI, BBS examinations, and without any medical history. The elderly participants must be 65 years of age or older, able to cooperate balance test, communicate with each other, and read words well. Exclusion criteria were severe somatic illness or neurological or musculoskeletal impairment including cognitive impairment, chest pain, angina pectoris, joint pain during recent exercise, congestive heart failure, and advised by doctors not to exercise. In all, 35 elderly adults (aged 82.06 ± 8.74 years) and 20 healthy young adults (aged 21.60 ± 0.60 years) participated. Postural control was measured according to the records of 15 joint coordinates. All participants were required to statically stand for 40 s while measurements were captured by a Kinect device. The recording procedure was performed daily for 6 days. The participants were instructed to stand and look straight at a visual reference and stand still with their shoulders relaxed, arms at the side of the trunk, feet slightly spread apart, and knee and hip joints in the upright position for 40 s. The participants were defined as young (control group) or elderly (experimental group) adults. The target class was the elderly group (experimental group) due to the lack of postural control. The experimental setup is depicted in Figure 1. All experimental procedures were approved by the Institutional Review Board of E-DA Hospital [with approval number EMRP-107-103 (2019/01/28)].

**Figure 2:**
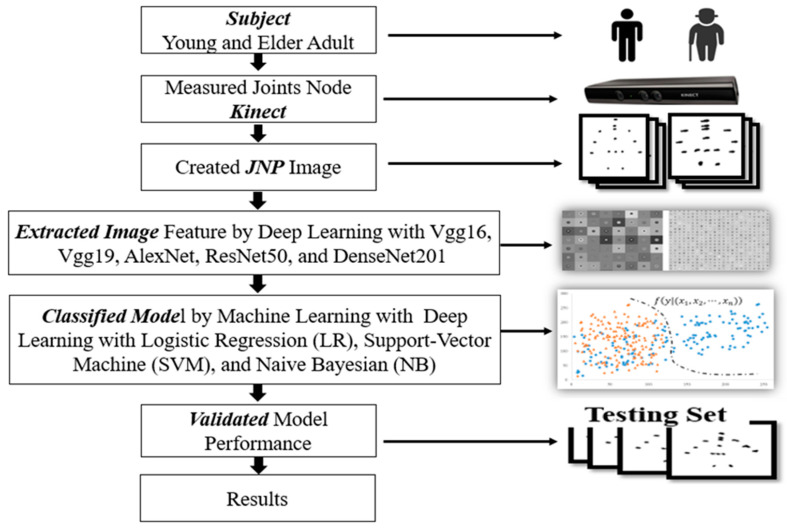
Study flowchart.

The study flowchart includes the participants coordinates of joint nodes measured by Kinect, creation of joint node images with the coordinates, features extracted from images, and training of the classification models. The models were validated with a testing set, and the final results were recorded (Figure 2).

### 2.2. Measurement of Joint Coordinates

The Kinect device was made by Microsoft (Microsoft Inc., Redmond, WA, USA). It recorded joint node locations and was connected to a personal computer–based signal processing system. A data point of a joint node signal includes *X*, *Y*, and *Z* coordinates. Only *X* and *Y* coordinates were considered in this study because when standing still, vertical movement is negligible. The signals of the joint nodes were recorded at a frequency of 30 Hz. 

### 2.3. Creating the JNP

**Figure 3:**
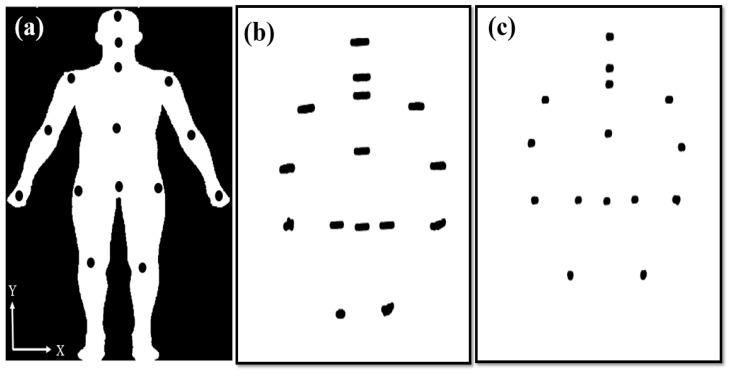
(**a**) The Kinect device recorded the positions of 15 joints; joint–node plots of (**b**) an elderly adults and (**c**) a young adult over a period of 40 s.

The 15 joints were recorded by Kinect for 40 s (Figure 3a). However, the 1200 coordinates (*X*, *Y*) of the joints were recorded over 40 s. Hence, the JNP was created to observe postural control and examine stability over a period of 40 s (Figure 3b,c). The JNPs clearly visualized good or poor postural control and provided positioning information for the deep and machine learning approaches.

### 2.4. Deep and Machine Learning Methods

Combinations of deep and machine learning methods were used to classify and predict postural control in young and elderly adults. The 90 model combinations involved five CNNs, three classifiers, three epochs (10, 15, and 20), and two random splitting ratios for the training set (60% and 70%) (i.e., 5  ×  3  ×  3  ×  2 combinations).

#### 2.4.1. Deep Learning Methods

The pre-trained CNNs applied to extract features of the JNPs were Vgg16, Vgg19, AlexNet, ResNet50, and DenseNet201. Deep CNN network technology has five primary layers: a convolutional layer, a pooling layer, a rectified linear unit layer, fully connected layers, and a softmax layer. The layers are listed in Table 2. The fully connected CNN layers extracted and stored the features of the input image. The used CNNs were described by Hsu et al. [40] (Table 2). The CNN has been confirmed to be efficient and useful for image feature extraction in the fields of biomedicine and biology [41,42,43]. Again, in the current study, the size of the epoch was set as 10, 15, or 20, and the training set percentage was 60% or 70% of data, randomly selected from the groups.

#### 2.4.2. Machine Learning Methods

Logistic regression (LR) is often applied to analyze associations between two or more predictors or variables. Regression analysis is commonly adopted to describe relations between predictors or variables to build a linear functional model, whereas regression modeling is usually used to predict an outcome with a new predictor. LR is a binary regression model. The LR method is used in the field of machine learning and is applied for the development of classification models because of its capacity to provide tree-like or hierarchical structures. Many fields have adopted LR for prediction and classification.

A support vector machine (SVM) is a supervised learning method with the ability to powerfully generate a Hyper Plan for classifying categorical data. The SVM is generally utilized in high-dimensional or nonlinear categorization. Many useful kernels are available to improve classification performance and reduce false rates. 

Naive Bayes (NB) classifiers are based on the Bayesian theorem with a naïve independence hypothesis between the adopted predictors or features. NB classifiers provide higher accuracy under bundle with kernel density estimation [44]. They also offer high flexibility for linear or nonlinear relations among variables (features/predictors) in classification problems. The computing cost takes linear time by compared those of expensive iterative approximations of classifiers. 

To classify the postural control of the young and elderly groups, these algorithms were applied to the extracted features as deep and machine learning methods with JNP.

### 2.5. Evaluating Model Performance

The coordinates of 15 joints continually measured for 40 s were plotted in one figure for each candidate. Each participant had six figures as a result of the replicated runs. Hence, a total of 120 and 150 JNPs were created for the young and elderly groups, respectively. The size of a JNP was 875 × 656 pixels with 24 bits per pixel. The testing sets were 48 and 60 JNPs (40%) or 36 and 45 JNPs (30%), randomly selected from the young and elderly groups, respectively. The original data were partitioned into training and testing sets randomly without overlapping samples in the sets.

The testing sets were used to evaluate model performance. The validated performance of the presented methods is typically used to popular index. A confusion matrix is often used to assess model suitability, including its accuracy, sensitivity, specificity, negative predictive value (NPV), positive predictive value (PPV), and kappa value. The indices (i.e., six evaluated values) were sorted in ascending order according to the kappa value. Then, a radar plot was developed to display the indexes for the models. A radar plot was developed to display those indexes for the presented models.

## 3. Results

### 3.1. Model Performance: 60% of Data for Training and 40% of Data for Testing

**Figure 4:**
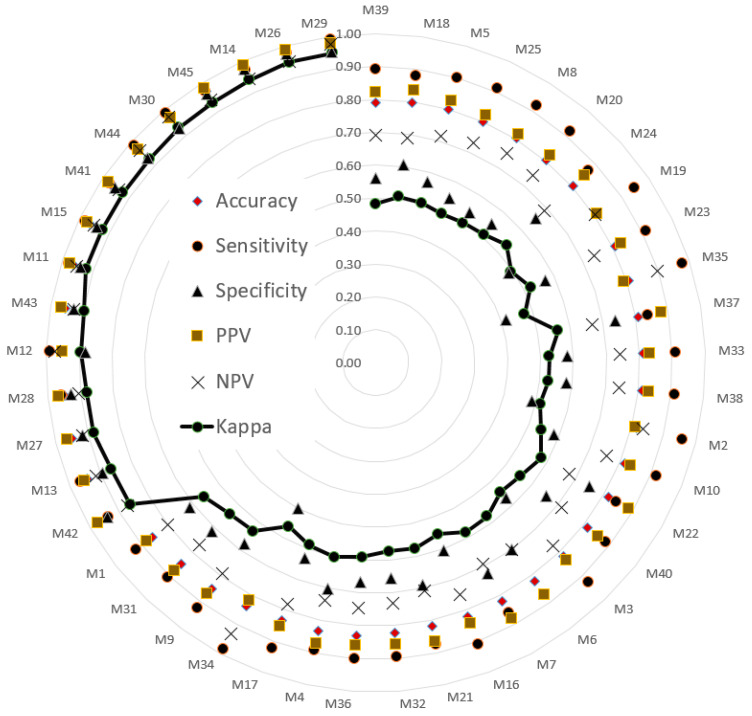
Performance of 45 models using 60% of the data as the training set. Model details are listed in Appendix A.

**Figure 5:**
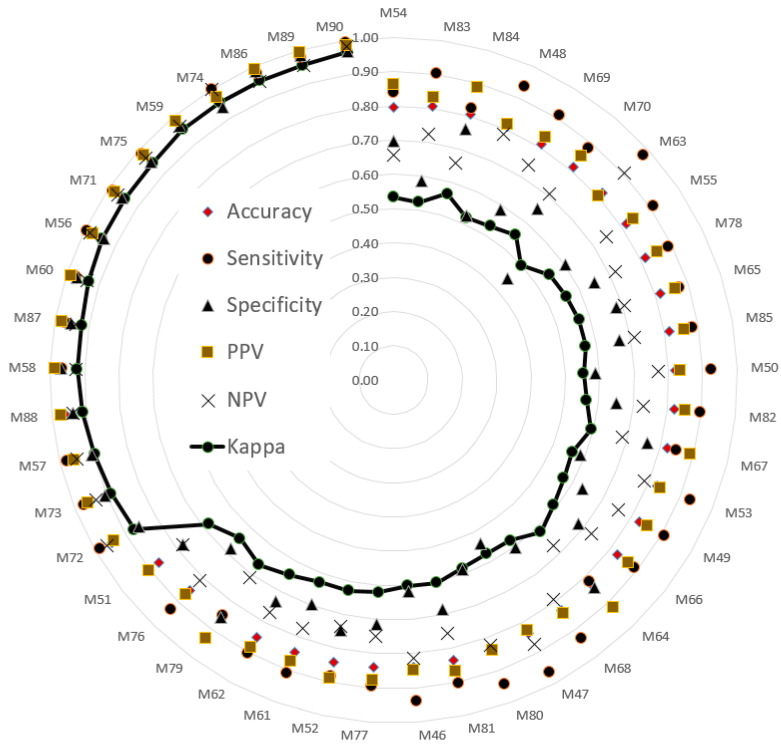
Performance of 45 models trained using 70% of the data. Model details are provided in Appendix A.

A total of 45 models were obtained according to five deep learning methods, with three batch sizes and three machine learning algorithms, and a 60% random splitting ratio of the original data. Another 45 models were obtained with a 70% random splitting ratio of the data. Figure 4 and Figure 5 present the validation results of testing sets with 40% and 30% random splitting ratios, respectively, of the original data.

The size of the epoch used in CNN models is crucial for making inferences regarding classification performance. Therefore, epoch size was considered during evaluation of the models. Figure 4 depicts the performance of 45 models (combinations) with 40% of the data used as the testing set. Table 3 details the performance of five CNNs. The model combining VGG16 and SVM under epoch 15 (M29) had the best performance out of all the models. The accuracy, sensitivity, specificity, PPV, NPV, and kappa value were 0.98, 0.99, 0.95, 0.98, 0.98, and 0.95, respectively. These results suggest that VGG16 extracted useful JNP features and the SVM feasibly classified the features. The SVM demonstrated high dimensional classification capacity and efficiency when combined with AlexNet, DenseNet201, ResNet50, VGG16, and VGG19, with an accuracy of 0.95 or higher generated by these combinations.

### 3.2. Model Performance: 70% of the Data Used for Training and 30% of the Data Used for Testing

Figure 5 portrays the performance of 45 models (combinations) when 70% and 30% of the data was used for training and testing, respectively. Table 4 presents the performance of five CNNs. The combination of VGG19 and SVM under epoch 20 (M90) had the best performance. The accuracy, sensitivity, specificity, PPV, NPV, and kappa value were 0.99, 0.99, 0.97, 0.99, 0.98, and 0.97, respectively. The second-highest performance was achieved by M89, which was a combination of VGG16 and SVM under epoch 20. Both VGG16 and VGG19 extracted useful JNP features, and the SVM feasibly classified them. The SVM demonstrated good classification ability and efficiency when combined with AlexNet, DenseNet201, ResNet50, VGG16, and VGG19, with accuracies of 0.95 or higher.

The combination of VGG19 and SVM with 70% of the data used for training in epoch 20 (M90) could be used to classify postural control in young and elderly groups through the JNP.

## 4. Discussion

### 4.1. The Informative JNP

Using joint motion trajectories instead of COP or COM displacement for analysis enables the evaluation of posture control ability as well as the posture control strategies used to achieve balance [45,46]. In the current study, the JNPs of the elderly group indicated that they tended to use an extreme joint coordination mode, an inter-joint coordination strategy characterized by total joint dependence, to maintain balance when standing still [19].

**Figure 6:**
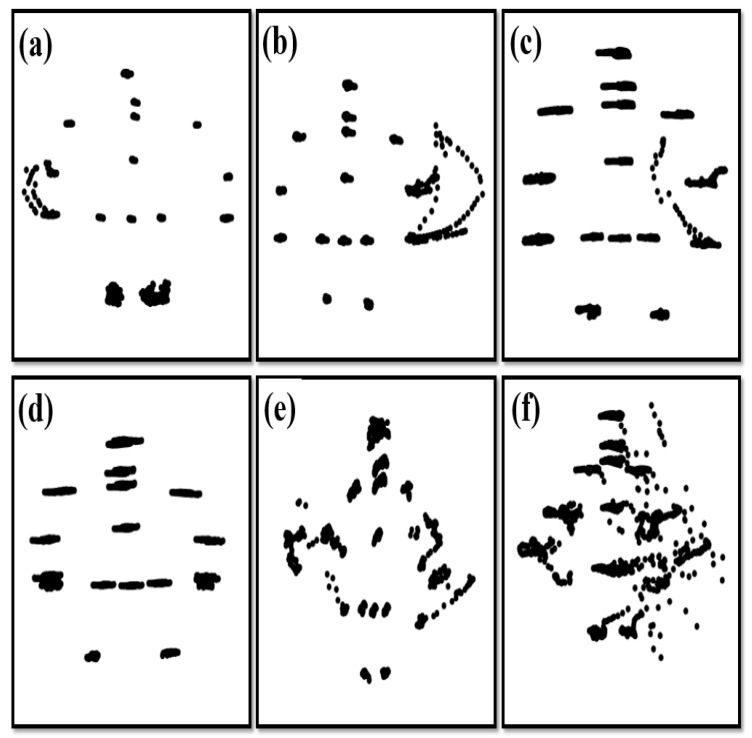
Abnormal patterns of postural control in several participants. (**a**) The forearm and knee joints exhibited slight tremors. (**b**) The forearm and hand joints exhibited obviously tremors. (**c**) The whole body shook horizontally and the left forearm shook more. (**d**) The whole body shook horizontally. (**e**) Symmetrical shaking of the wrists and lower limbs occurs on both sides. (**f**) Individual stands with whole-body asymmetrical shaking.

The JNP provided information on postural control, but also on tremors. No screening test or tool is available for the early detection of Parkinson’s disease. The JNP map can help in evaluating coordinated interactions among joints and discovering involuntary tremors of each segment when an individual is standing still [47]. The stability of the torso and proximal joints in the elderly adult group was similar to that in the young adult group, but the forearm and knee joints exhibited slight tremors (Figure 6a), which may have been psychogenic or physiological tremors. Figure 6b displays the postural stability of joints in various parts of the body, which was better than most of elderly people in the study but the forearm and hand joints exhibited obviously psychogenic or physiological tremors. The postural stability of the joints of various parts of the body in Figure 6d is similar to that in Figure 6c and may indicate a postural tremor. In some cases, the left forearm shook more, but the whole body shook horizontally (Figure 6c). Figure 6c displayed a typical pattern of postural stability of the joints in the elderly adult group and possibly indicating a postural tremor and or psychogenic tremor. When a postural tremor occurs, further testing is required to confirm its cause, which may be, for example, primary cerebellar disease, brain injury, dystonia, alcohol, or drugs. In Figure 6e, symmetrical shaking of the wrists and lower limbs occurs on both sides; this is suspected to be an essential tremor or Parkinsonian tremor. In Figure 6f, whole-body shaking, including shaking of the feet, is intense and asymmetrical and leads to instability when the individual is standing; in such cases, a Parkinsonian tremor is suspected. When a postural tremor occurs in a case, further testing is required to confirm the cause of the jitter, which may be caused by other diseases, such as primary cerebellar disease, dystonia, Parkinson’s disease, drugs, etc. Hence, the JNP may be used to visualize shaking and relations between tremors and diseases.

### 4.2. Combined Deep and Machine Learning

In this study, the VGG16, VGG19, AlexNet, ResNet50, and DenseNet201 were used to extract image features for the development of SVM classification models. Although several fully connected layers (FCLs) were present in the CNN, we did not survey and compare all of them. Only the last FCL of the CNN was applied to extract features of images for the SVM, LR, and NB classifiers. The SVM was regarded as an efficient classifier for detecting and classifying postural control in young and elderly adults on the basis of their JNPs. 

M29 combined VGG16 and the SVM (training set, 70%; validation set, 30%), and M90 combined VGG19 and SVM to classify balance function (training set, 80%; validation set, 20%). The accuracy and kappa values of M29 and M90 were (98%, 95%) and (99%, 97%), respectively. The validation results indicated that both M29 and M90 could classify balance function in the elderly group with high agreement and consistency. Additionally, the deep learning component of the VGG architecture provided useful features of images for the SVM. Therefore, the SVM archived to classify the task of detected balance function between the young and elderly adults.

Table 5 summarizes the results in Table 3 and Table 4. All 27 methods selected achieved kappa values of 0.88 or higher. AlexNet, VGG16, and VGG19 each appeared six times. DenseNet201 and ResNet50 appeared four and five times, respectively. The minimum accuracy generated by AlexNet, VGG16, and VGG19 was 0.97. VGG19 combined with the SVM had the highest maximum accuracy among the five deep learning methods with the SVM classifier.

### 4.3. Comparison with Reported Results

The proposed methods were compared with previously developed methods with respect to the results listed in Table 5. SVMs, random forest models, and cohorts have been applied to detect motor [48,49], balance, or gait function [50,51,52,53,54,55,56,57,58,59,60]. The highest accuracy in classifying motor function was 97%, achieved by an SVM. The highest accuracy for classifying gait or balance function was 96.7%, also achieved by an SVM. Thus, SVMs were proven successful in classification tasks. However, the proposed methods achieved higher accuracies in terms of reasonability and feasibility than did the other methods listed in Table 6. 

To further test the reliability of the proposed methods in classifying postural control, a future study might compare the results a gold standard detection method, such as functional assessment or balance assessment.

## 5. Conclusions

The JNP can reveal postural coordinates in a two-dimensional image. Moreover, it provides visual information on postural swing and is suitable for the classification and detection of postural control in young and elderly adults when used with the deep and machine learning methods developed in this study. The best performance was achieved through the combination of VGG19 and SVM with 70% of the data used for the training set and an epoch of 20. The correlations between JNPs and clinical tremors can be investigated in future research. Indeed, both the elderly and the young group should be screened or some gold standard detection (e.g., psychologist, radiologist, or related medical assessor) be applied to verify the reality of both groups. In this study, the lack of a serious screening test or use of a gold standard detection method for each subject is noted as a limitation of the research. Future work can incorporate more rigorous screening.

## Figures and Tables

**Table 1:** Demographic characteristics of the samples (N = 35 + 20 = 55).

Index	Elderly (n = 35)		Young (n = 20)	
	Mean	SD	Mean	SD
**Age**	80.17	8.56	20.00	1.97
**MMSE**	24.91	3.40	30.00	0.00
**BI**	86.57	4.16	100.00	0.00
**BBS**	47.09	6.47	56.00	0.00

**Table 2:** Pre-trained models used in this study [40].

Pre-Trained Model	Input Image Size	Design Layers	Parametric Size (MB)	Layer of Features
AlexNet	227 × 227	25	227	17th
DenseNet201	224 × 224	709	77	706th
ResNet50	224 × 224	177	96	175th
VGG16	224 × 224	41	27	33rd
VGG19	224 × 224	47	535	39th

**Table 3:** Models trained with 60% of the data had accuracy and kappa values of no less than 0.95 and 0.88, respectively.

Model	Epoch	CNN	Learner	Accuracy	Sensitivity	Specificity	PPV	NPV	Kappa
M27	15	DenseNet201	SVM	0.95	0.96	0.92	0.96	0.92	0.88
M28	15	ResNet50	SVM	0.95	0.96	0.93	0.97	0.91	0.88
M12	10	DenseNet201	SVM	0.96	0.99	0.88	0.95	0.97	0.90
M43	20	ResNet50	SVM	0.96	0.97	0.93	0.97	0.93	0.90
M11	10	AlexNet	SVM	0.97	0.98	0.94	0.97	0.95	0.92
M15	10	VGG19	SVM	0.97	0.98	0.94	0.97	0.95	0.92
M41	20	AlexNet	SVM	0.97	0.97	0.95	0.98	0.94	0.92
M44	20	VGG16	SVM	0.97	0.98	0.93	0.97	0.96	0.92
M30	15	VGG19	SVM	0.97	0.99	0.93	0.97	0.98	0.93
M45	20	VGG19	SVM	0.97	0.97	0.96	0.98	0.94	0.93
M14	10	VGG16	SVM	0.98	0.97	0.98	0.99	0.94	0.94
M26	15	AlexNet	SVM	0.98	0.98	0.98	0.99	0.95	0.95
M29	15	VGG16	SVM	0.98	0.99	0.95	0.98	0.98	0.95

**Table 4:** Models trained with 70% of the data achieved accuracy and kappa values of no less than 0.95 and 0.88, respectively.

Model	Epoch	CNN	Learner	Accuracy	Sensitivity	Specificity	PPV	NPV	Kappa
M73	15	ResNet50	SVM	0.95	0.97	0.91	0.96	0.93	0.89
M57	10	DenseNet201	SVM	0.96	0.98	0.91	0.96	0.95	0.90
M88	20	ResNet50	SVM	0.96	0.97	0.94	0.97	0.94	0.91
M58	10	ResNet50	SVM	0.97	0.97	0.97	0.99	0.92	0.92
M87	20	DenseNet201	SVM	0.97	0.97	0.95	0.98	0.94	0.92
M60	10	VGG19	SVM	0.97	0.97	0.97	0.99	0.94	0.93
M56	10	AlexNet	SVM	0.98	0.99	0.94	0.97	0.98	0.94
M71	15	AlexNet	SVM	0.98	0.99	0.95	0.98	0.97	0.94
M75	15	VGG19	SVM	0.98	0.99	0.95	0.98	0.97	0.94
M59	10	VGG16	SVM	0.98	0.99	0.97	0.99	0.97	0.96
M74	15	VGG16	SVM	0.98	1.00	0.94	0.97	1.00	0.96
M86	20	AlexNet	SVM	0.98	0.98	0.98	0.99	0.95	0.96
M89	20	VGG16	SVM	0.98	0.98	0.98	0.99	0.95	0.96
M90	20	VGG19	SVM	0.99	0.99	0.97	0.99	0.98	0.97

**Table 5:** Summary of the results in Table 3 and Table 4.

Deep Learning	Counts	Min. ACC	Max. ACC
AlexNet	6	0.97	0.98
DenseNet201	4	0.95	0.97
ResNet50	5	0.95	0.97
VGG16	6	0.97	0.98
VGG19	6	0.97	0.99
Total	27		

Note: Min. ACC and Max. ACC are the minimum and maximum accuracy.

**Table 6:** Comparison of the proposed methods with methods developed in related studies.

Author	Year	Methods	Task	Sample Size	Performance
Di Lazzaro G. et al. [48]	2020	SVM	motor	65	ACC: 97% (SVM)
Yuhan Zhou et al. [50]	2020	SVM	gait	239	ACC: 89% (SVM)
		RF			ACC: 73% (RF)
		ANN			ACC: 90% (ANN)
Tian Bao et al. [53]	2019	SVM	balance	16	ACC: 82% (SVM)
Jianwei Niu et al. [56]	2019	SVM	gait	12	ACC: 96.7% (SVM)
Narintip Roongbenjawan et al. [59]	2020	Cohort Study	balance	73	SEN: 92%
					SPE: 81%
The Presented Methods	2021	DL + ML	balance	55	ACC: 98% (VGG16 + SVM)
					ACC: 99% (VGG19 + SVM)

Note: ACC is accuracy. SPE is specificity. RF is random forest. SVM is support vector machine. DL is deep learning. ML is machine learning.

## Data Availability

Not applicable.

## Appendix A

The 90 investigated methods with combined CNNs and classifiers; the percentage of data used for the training set and the epoch size are listed.

**Epoch**	**Ratio**	**CNN**	**Learner**	**Model**	**Epoch**	**Ratio**	**CNN**	**Learner**	**Model**
10	0.6	AlexNet	LR	M1	15	0.7	AlexNet	LR	M46
10	0.6	DenseNet201	LR	M2	15	0.7	DenseNet201	LR	M47
10	0.6	ResNet50	LR	M3	15	0.7	ResNet50	LR	M48
10	0.6	VGG16	LR	M4	15	0.7	VGG16	LR	M49
10	0.6	VGG19	LR	M5	15	0.7	VGG19	LR	M50
10	0.6	AlexNet	NB	M6	15	0.7	AlexNet	NB	M51
10	0.6	DenseNet201	NB	M7	15	0.7	DenseNet201	NB	M52
10	0.6	ResNet50	NB	M8	15	0.7	ResNet50	NB	M53
10	0.6	VGG16	NB	M9	15	0.7	VGG16	NB	M54
10	0.6	VGG19	NB	M10	15	0.7	VGG19	NB	M55
10	0.6	AlexNet	SVM	M11	15	0.7	AlexNet	SVM	M56
10	0.6	DenseNet201	SVM	M12	15	0.7	DenseNet201	SVM	M57
10	0.6	ResNet50	SVM	M13	15	0.7	ResNet50	SVM	M58
10	0.6	VGG16	SVM	M14	15	0.7	VGG16	SVM	M59
10	0.6	VGG19	SVM	M15	15	0.7	VGG19	SVM	M60
10	0.7	AlexNet	LR	M16	20	0.6	AlexNet	LR	M61
10	0.7	DenseNet201	LR	M17	20	0.6	DenseNet201	LR	M62
10	0.7	ResNet50	LR	M18	20	0.6	ResNet50	LR	M63
10	0.7	VGG16	LR	M19	20	0.6	VGG16	LR	M64
10	0.7	VGG19	LR	M20	20	0.6	VGG19	LR	M65
10	0.7	AlexNet	NB	M21	20	0.6	AlexNet	NB	M66
10	0.7	DenseNet201	NB	M22	20	0.6	DenseNet201	NB	M67
10	0.7	ResNet50	NB	M23	20	0.6	ResNet50	NB	M68
10	0.7	VGG16	NB	M24	20	0.6	VGG16	NB	M69
10	0.7	VGG19	NB	M25	20	0.6	VGG19	NB	M70
10	0.7	AlexNet	SVM	M26	20	0.6	AlexNet	SVM	M71
10	0.7	DenseNet201	SVM	M27	20	0.6	DenseNet201	SVM	M72
10	0.7	ResNet50	SVM	M28	20	0.6	ResNet50	SVM	M73
10	0.7	VGG16	SVM	M29	20	0.6	VGG16	SVM	M74
10	0.7	VGG19	SVM	M30	20	0.6	VGG19	SVM	M75
15	0.6	AlexNet	LR	M31	20	0.7	AlexNet	LR	M76
15	0.6	DenseNet201	LR	M32	20	0.7	DenseNet201	LR	M77
15	0.6	ResNet50	LR	M33	20	0.7	ResNet50	LR	M78
15	0.6	VGG16	LR	M34	20	0.7	VGG16	LR	M79
15	0.6	VGG19	LR	M35	20	0.7	VGG19	LR	M80
15	0.6	AlexNet	NB	M36	20	0.7	AlexNet	NB	M81
15	0.6	DenseNet201	NB	M37	20	0.7	DenseNet201	NB	M82
15	0.6	ResNet50	NB	M38	20	0.7	ResNet50	NB	M83
15	0.6	VGG16	NB	M39	20	0.7	VGG16	NB	M84
15	0.6	VGG19	NB	M40	20	0.7	VGG19	NB	M85
15	0.6	AlexNet	SVM	M41	20	0.7	AlexNet	SVM	M86
15	0.6	DenseNet201	SVM	M42	20	0.7	DenseNet201	SVM	M87
15	0.6	ResNet50	SVM	M43	20	0.7	ResNet50	SVM	M88
15	0.6	VGG16	SVM	M44	20	0.7	VGG16	SVM	M89
15	0.6	VGG19	SVM	M45	20	0.7	VGG19	SVM	M90
